# Physical activity in Sami and non-Sami populations in rural Northern Norway, the SAMINOR 2 Clinical Survey

**DOI:** 10.1186/s12889-021-11744-2

**Published:** 2021-09-14

**Authors:** Kristin Benjaminsen Borch, Bent Martin Eliassen, Marita Melhus, Elin Damsgård, Ann Ragnhild Broderstad

**Affiliations:** 1 Department of Community Medicine, Faculty of Health Sciences, UiT The Arctic University of Norway, Tromsø, Norway; 2 Faculty of Nursing and Health Sciences, Nord University, Bodø, Norway; 3 Centre for Sami Health Research, Department of Community Medicine, Faculty of Health Sciences, UiT The Arctic University of Norway, Tromsø, Norway; 4 Department of Health and Care Sciences, Faculty of Health Sciences, UiT The Arctic University of Norway, Tromsø, Norway

**Keywords:** Physical activity, Indigenous health, Sami, Norwegian, SAMINOR, Survey

## Abstract

**Background:**

The Sami people is an indigenous minority population living in the northern parts of Norway and mainly in rural areas. We lack data of contemporary levels of physical activity (PA) in rural regions of Northern Norway and in the Sami population in particular. We aimed to describe the PA levels and investigate whether PA levels differs between Sami and non-Sami and between coastal and inland areas.

**Methods:**

We used data from the second survey of the Population-based Study on Health and Living Conditions in Regions with Sami and Norwegian Populations – the SAMINOR 2 Clinical Survey (2012–2014) that includes the adult population in 10 municipalities in the counties Troms, Finnmark and Nordland. Participants self-reported on PA, ethnicity and modifiable lifestyle factors. Twelve thousand four hundred fifty-five individuals were invited with a response rate of 48.2% (*n* = 6004 participants). We tested differences using chi-square tests, two sample t-tests and linear regression models.

**Results:**

Among 5628 participants, 41.1 and 40.9% of men and women, respectively, were defined as Sami. We found no ethnic differences in PA in men overall. However, Sami men living in Tana, and Nesseby reported higher PA compared to non-Sami men in the same area. For Sami women there was overall lower PA levels compared to non-Sami women, especially pronounced in Kautokeino/ Karasjok.

**Conclusion:**

This study showed small differences in PA levels between Sami and non-Sami men. Sami women had lower PA levels compared to their non-Sami counterparts. It is important to identify whether there are differences in various ethnic populations, together with other predictors for PA in future planning of public health interventions.

## Introduction

Strong evidence shows that physical inactivity and sedentary behaviour increase the risk of several health conditions, including non-communicable diseases such as coronary heart disease, type 2 diabetes, some cancers, depression and risk of falls [1, 2]. This represents a high burden of disease for the society, shortens life expectancy and is a major threat to public health [1]. Insufficient physical activity (PA) level is one of ten leading risk factors for global mortality [3]. Data from the World Health Organization (WHO) 2018 show that one in four adults do not meet the recommendations of PA to benefit from reduced risk of non-communicable diseases, and only 23% of men and 32% of women being sufficiently physically active [4]. In Norway, two national surveys is reported on PA levels among adults [5, 6]. In the first survey (2008/2009), 20.4% of adults met the national recommendation for PA corresponding to 150 min/week of moderate/vigorous PA, with no differences among men and women [7]. In the second survey (2014/2015) the prevalence was estimated to 34% for women and 29% for men [8]. The study included 5099 Norwegian men and women in a nationally representative sample and PA level was collected using accelerometer based data [5]. However, this study had a limited number of persons with non-Norwegian origin, nor did the study sample specify whether other ethnic groups were represented [5].

The Sami people is an indigenous minority population living in the northern parts of Norway, Sweden and Finland, and Russia’s Kola Peninsula. Even though the general health of the Sami people in Norway do not differ substantially from that of the majority population, some differences have been demonstrated [9]. Higher prevalence of type 2 diabetes mellitus and higher obesity indices have been observed among Sami relative to non-Sami counterparts in the SAMINOR study, together with metabolic syndrome in women only [10–15]. Furthermore, there have been observed a higher risk of stroke, and Sami people have reported symptoms of angina pectoris more frequently compared to non-Sami [15, 16]. For cancer incidence among the Sami population in Norway, a follow-up study for the years 1970–1997 showed a lower incidence of colon, lung, breast and prostate cancer, higher incidence of oesophageal cancer among men and no differences for other cancer sites when compared to the reference population [17]. In Norway, all inhabitants, including the Sami people, have equal access to health services, however the quality varies caused by language barriers and lack of cultural knowledge [18, 19]. A study published recently on changes in self-reported leisure-time PA among adults in Finnmark, Northern Norway, showed that the total proportion of sedentary individuals during leisure-time, decreased between 1987 and 2003. However, the proportion of sedentary individuals was higher in Sami than in non-Sami, at both baseline and at the end of follow-up [20]. At the same time, both Sami men and women reported significantly more occupational physical activity levels [21].

Knowledge regarding the contemporary levels of PA in rural regions of Northern Norway is lacking, and especially with regard to the Sami population herein. To our knowledge there are few other published studies on PA levels conducted in rural areas inhabited with Sami population after 2003 [20, 21]. The aim of this study was to describe the self-reported PA levels in the Sami and non-Sami population in rural areas in Northern Norway, and assess whether there are differences in PA levels according to ethnicity, geography and gender. We used data from the second survey of the Population-based Study on Health and Living Conditions in Regions with Sami and Norwegian Populations – The SAMINOR 2 Clinical Survey 2012–2014 [22].

## Methods

### Study participants

**Fig. 1:**
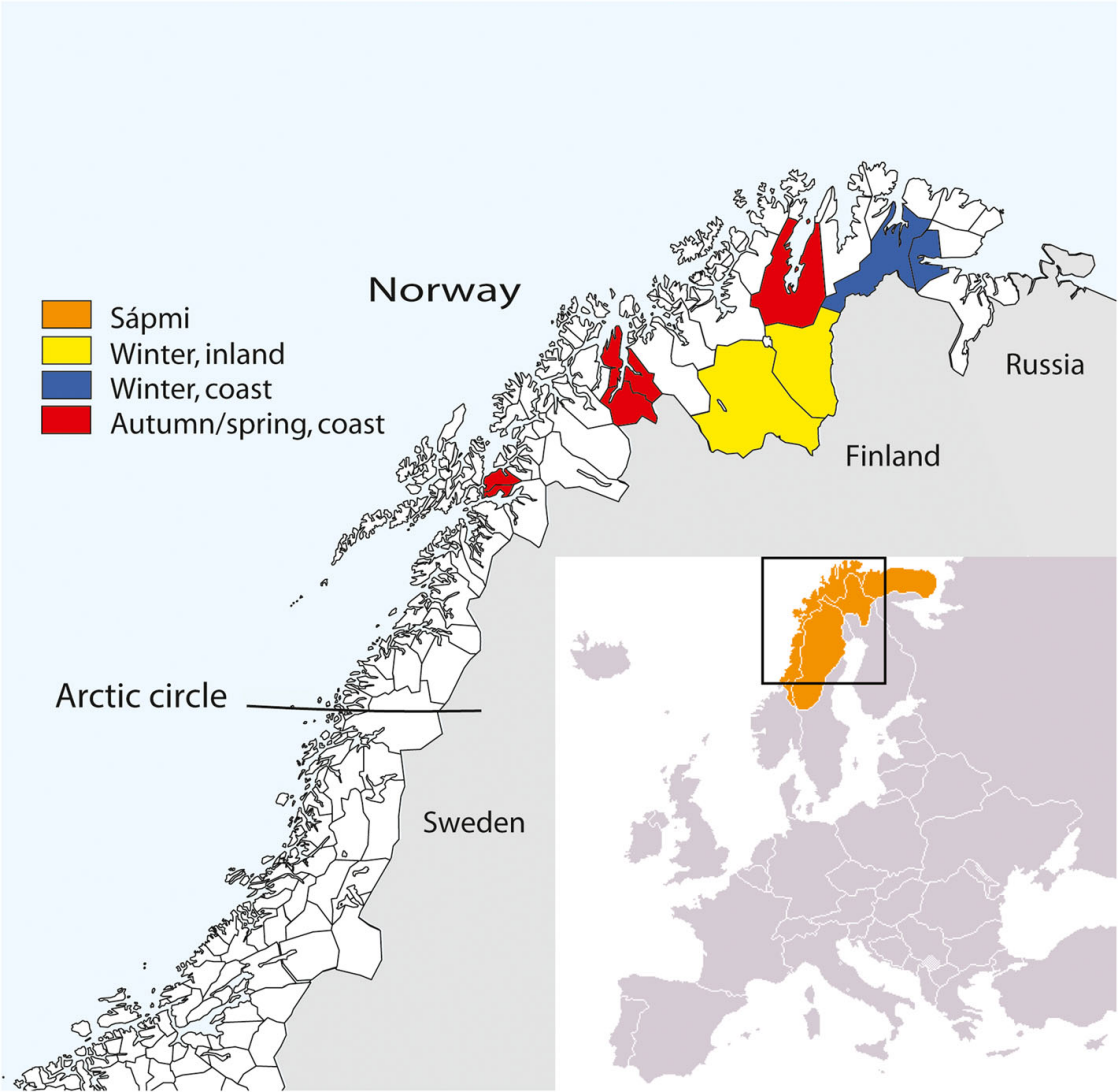
Map of Northern Norway, Sápmi and the included municipalities in the SAMINOR 2

The present study used data from the SAMINOR 2 Clinical Survey (hereinafter entitled SAMINOR 2), which was performed in 2012–2014 [22]. The survey included 10 municipalities in Finnmark, Troms and Nordland: Nesseby, Tana, Porsanger, Karasjok Kautokeino, Kåfjord, Storfjord, Lyngen, Skånland and Evenes (Fig. 1). The municipalities have from < 1000 to 4000 inhabitants and are populated by both Sami and non-Sami people. All municipalities are within the Northern Sami language area. The total population of inhabitants aged 40–79 in the selected municipalities were eligible, regardless of their ethnic background. They were identified through the Norwegian National Population Register and received a written invitation to participate in the study [22]. In total, 12,455 individuals were invited and 6004 participated, which gives an overall response rate of 48.2%. We excluded 376 participants due to missing information on physical activity or ethnicity, leaving 5628 individuals in the analytical sample.

### Ethics

All participants gave written informed consent to participate in the study. The project group adheres to the Helsinki Declaration. SAMINOR 2 has been assessed by the Regional Committee for Medical and Health Research Ethics, North (2011/1840 and 2017/147) and the Norwegian Data Protection Authority (ref: 02/01525–4) and together with the SAMINOR Project Board, all have approved the study. The SAMINOR Study is designed to study the health and living conditions of the Sami population in Norway and is run by the Centre for Sami Health Research. The project adheres to ethical guidelines for Sami Health Research.

### Data collection

Data collection was performed in one municipality at a time. The period of data collection was 2–7 weeks in each municipality depending on the population size, and varied over different seasons of the year. Data was collected through clinical investigations and an 8-page paper questionnaire filled in by each participant. The questionnaire was available in Norwegian and Northern and Northern Sami. In two municipalities, only the Norwegian version was available. In four municipalities, invitees received questionnaires in both languages, and in four others, the Sami questionnaire was available upon request. This was due to varying numbers of Sami speaking inhabitants in the different municipalities. The questionnaire included information on living conditions, health, ethnicity, physical activity and other lifestyle factors. Details of the data collection are described elsewhere [22].

### Self-reported physical activity levels

PA levels were assessed at enrolment on an ordinal 10-point scale after reading the following explanation: *“By physical activity we mean activity both at work and outside work, at home, as well as training/exercise and other physical activity, such as walking, etc. Please mark the number that best describes your level of physical activity; 1 being very low and 10 being very high”.* The scale therefore reflects the amount of PA across different domains, including recreational, occupational, transportation, and household PA, and combines them into one global assessment of the PA level. This PA scale has been used in the Norwegian Women and Cancer Study [23], and has been validated among women aged 40–55 years living in Tromsø, Norway, assessed with an objective method combining accelerometer and heart rate sensor [24]. It appeared valid to rank the PA level in Norwegian women (i.e. Spearman’s rank correlation coefficient in the range of 0.36–0.46) and is comparable with other self-reported methods against objective methods, but without providing data on frequency, duration, intensity or domain of PA [24].

### Self-reported ethnicity

We measured ethnicity by the following question: “*What language(s) do/did you, your parents and your grandparents use at home (You may choose one or more alternatives: “Norwegian”, “Sami”, “Kven”, or “other”)?”* Providing the same response options, we asked the participants “*What is your, your father’s and your mother’s ethnic background?”* and finally, “*What do you consider yourself to be?”* Kvens are descendants of Finnish speaking settlers who immigrated to northern Norway in the 1700s and 1800s [25]. The following two criteria were used to classify Sami ethnicity; self-identification as a Sami by answering Sami as their own ethnic background or that they consider themselves Sami and, in addition, that at least one of the grandparents, parents or themselves had Sami as their domestic language. The criteria of both self-definition and language connection resembles the same criteria used by the Sami Parliament to be eligible to vote or be elected to the Parliament. We categorized all participants who did not meet these criteria as non-Sami.

### Height and weight measurements

We measured height and weight using an electronic Height, Weight & Fatness Measuring System device (DS-103, Dongsahn Jenix, Seoul, Korea). The participants were wearing light clothes without shoes. The body mass index (BMI) was calculated as weight in kg divided by the square of height in meters (kg/m^2^).

### Other information

The participants reported years of education, smoking status, and alcohol intake, chronic diseases including cardiovascular diseases (CVD, comprising myocardial infarction, angina pectoris, atrial fibrillation, heart (bypass) surgery, stent placement and/or use of antihypertensive drugs), diabetes, and chronic pain that had lasted 3 months or more. We obtained information on age, sex and municipality from the Norwegian National Population Register.

### Statistical analyses

The included sample characteristics were BMI (< 25, 25–29.9, ≥30 kg/m^2^), smoking habits (never, former, current smokers), alcohol consumption during the past year (none, < 2, 2–4.99, 5–9.99, ≥10 g/day), self-reported CVD was combined into “total CVD” (yes/no), and diabetes (yes/no), chronic pain (yes/no), education attainment (< 13/≥13 years). Missing values on anti-hypertensive medication use, myocardial infraction, angina pectoris, heart surgery, atrial fibrillation, diabetes, and chronic pain the last 3 months was considered negative responses. Those claiming being disease free in terms of myocardial infraction, angina pectoris, and diabetes, but reporting their age at disease onset, was considered positive cases, respectively. As physical activity levels may be impacted by climate, nature, local facilities and culture, the ten municipalities were categorized into three groups according to geographical location (coast-inland) and season of data collection as: “winter, inland” (Kautokeino and Karasjok), “winter, coast” (Tana and Nesseby) and “autumn/spring, coast” (Porsanger, Kåfjord, Storfjord, Lyngen, Skånland and Evenes) (Fig. 1). The three groups also differ in ethnic composition.

The statistical analyses included crude sample characteristics for Sami and non-Sami women, and Sami and non-Sami men, respectively. PA was categorized in five categories of PA level (1–2, 3–4, 5–6, 7–8, and 9–10) and presented by sex, ethnicity and ten-year age groups. Further, we calculated the age-standardised distribution of PA (direct standardisation with the invited sample in 10-year age groups as standard). Mean PA levels are presented by sex, ethnicity, age groups and geographical regions. Ethnic differences were examined by chi-square and two-sample t-tests, whenever appropriate.

Linear regression was used to test for Sami vs non-Sami differences in PA while adjusting for relevant variables, treating PA as a continuous variable (all ten categories). In model 1, we adjusted for age, only. In model 2, we also adjusted for education (< 13/≥13 years), smoking (never/former/current), alcohol intake (none, < 2, 2–4.99, 5–9.9, ≥10 g/day), cardiovascular diseases (yes/no), diabetes (yes/no), chronic pain (yes/no) and body mass index (< 25, 25–29.9, ≥30 kg/m^2^). All statistical analyses were performed using Stata V.15.0 (StataCorp, College Station, TX). All tests were two-sided with a 5% significance level.

## Results

We identified 40.9 and 41.1% of women and men, respectively, as Sami (Tables 1 and 2). The proportion of Sami differed by region; the Sami were in large majority in the “winter, inland” group, and a smaller majority in the “winter, coast” group, while they were in minority in the “autumn, spring, coast” group (Tables 1 and 2). Furthermore, Table 1 shows that there was no overall age difference between Sami and non-Sami women. We observed statistically significant differences (*p*-values are presented in Table 1) for the following characteristics; The Sami women situated in coastal areas with data collection in the wintertime (Tana/Nesseby) were on average older than the non-Sami women were (59.2 versus 56.9). Compared to non-Sami women, Sami women reported less chronic pain (42.5% versus 48.5%), a higher proportion (33.2% versus 27.0%) was classified as obese (BMI ≥30 kg/m2), and a higher proportion reported higher educational level (48.7% versus 44.6%). Alcohol intake past year was significantly lower in Sami women versus the non-Sami counterparts. Furthermore, investigating these characteristics by region/season and ethnic groups the differences were less prominent. In the Sami women at inland regions (Kautokeino/Karasjok) with data collection in the winter, obesity and low alcohol intake were more prominent. Compared to the non-Sami women, there were a slightly higher proportion of current smokers among Sami women, however not statistically significant. In Table 2, the descriptive characteristics showed no overall age difference between Sami and non-Sami men, but in Tana/Nesseby the Sami men were on average slightly older than the non-Sami men (61.6 versus 59.6) (*p*-values are presented in Table 2). Among Sami men, a lower proportion reported CVD (34.3% versus 38.8%) and a higher proportion had obesity compared to their non-Sami counterparts (31% versus 27%). Education level differed significantly between Sami and non-Sami men in the inland areas (35.7% versus 53.1%), whereas this difference was less prominent in the total sample of men. Lastly, Sami men had a lower alcohol intake compared to non-Sami men.

**Table 1:** Characteristics for women, by region/season and ethnic group. The SAMINOR 2 Clinical Survey (2012–2014)

	Winter, inland^b^			Winter, coast^c^			Autumn/spring, coast^d^			Total		
	Sami	Non-Sami	*P*-value	Sami	Non-Sami	*P*-value	Sami	Non-Sami	*P*-value	Sami	Non-Sami	*P*-value
Number^a^	590	97		281	215		359	1466		1230	1778	
Mean age (SD) (years)	57.7 (10.4)	58.3 (10.4)	0.62	59.2 (10.4)	56.9 (10.8)	0.02	58.2 (10.1)	58.8 (10.5)	0.34	58.2 (10.3)	58.5 (10.6)	0.37
Education (years)			< 0.001			0.62			0.004			0.03
< 13	53.0	25.0		49.4	47.1		50.0	58.5		51.3	55.4	
≥ 13	47.0	75.0		50.6	52.9		50.0	41.5		48.7	44.6	
Smoking			0.02			0.60			0.10			0.23
Never	41.9	54.2		35.1	32.6		34.9	39.9		38.3	39.8	
Former	35.6	34.4		44.9	43.9		40.9	40.3		39.3	40.4	
Current	22.5	11.5		19.9	23.6		24.2	19.8		22.4	19.8	
Alcohol past year (g/day)			< 0.001			0.40			0.36			< 0.001
No alcohol	48.2	27.8		25.5	26.0		25.8	26.1		36.0	26.2	
< 2.0	35.2	32.0		40.1	32.7		33.2	33.7		35.7	33.2	
2.0–4.99	9.6	15.5		14.3	17.8		17.1	17.3		13.0	17.2	
5.0–9.99	6.3	17.5		15.1	19.2		15.2	17.1		11.1	17.4	
≥ 10.0	0.7	7.2		5.0	4.3		8.7	5.8		4.2	5.7	
Diseases												
CVD	28.3	26.8	0.76	31.3	29.8	0.71	32.3	32.5	0.95	30.2	31.8	0.33
Diabetes	8.0	4.1	0.18	5.3	3.7	0.40	8.4	8.4	0.98	7.5	7.6	0.91
Chronic pain	41.7	44.3	0.63	38.4	42.3	0.38	47.1	49.7	0.37	42.5	48.5	0.001
BMI categories (kg/m^2^)			< 0.001			0.18			0.59			< 0.001
< 25	23.4	33.3		26.0	32.7		30.3	32.9		26.0	32.9	
25–29.9	37.8	49.0		45.2	37.9		42.3	39.8		40.8	40.1	
≥ 30	38.8	17.7		28.8	29.4		27.5	27.3		33.2	27.0	

*SD* standard deviation, *CVD* Cardiovascular disease (Myocardial infarction, angina pectoris, atrial fibrillation, heart (bypass) surgery, stent placement and/or use of antihypertensive drugs), *BMI* body mass index

^a^Subgroups may not total to this number due to missing values

^b^Municipalities included in winter, inland area: Kautokeino, Karasjok

^c^Municipalities included in winter, coast area: Tana and Nesseby

^d^Municipalities included in autumn/spring, coast area: Lyngen, Porsanger, Skånland, Evenes, Storfjord and Kåfjord

**Table 2:** Characteristics for men, by region/season and ethnic group. The SAMINOR 2 Clinical Survey (2012–2014)

	Winter, inland^b^			Winter, coast^c^			Autumn/spring, coast^d^			Total		
	Sami	Non-Sami	*P*-value	Sami	Non-Sami	*P*-value	Sami	Non-Sami	*P*-value	Sami	Non-Sami	*P*-value
Number^a^	448	67		282	186		347	1290		1077	1543	
Mean age (SD) (years)	58.2 (10.1)	58.8 (10.5)	0.70	61.6 (10.6)	59.6 (9.5)	0.04	60.0 (9.9)	60.3 (10.3)	0.66	59.7 (10.3)	60.2 (10.2)	0.26
Education (years)			0.01			0.86			0.34			0.06
< 13	64.3	46.9		68.4	67.6		64.6	61.7		65.5	61.8	
≥ 13	35.7	53.1		31.6	32.4		35.4	38.3		34.5	38.2	
Smoking			0.08			0.11			0.12			0.19
Never	28.7	41.8		33.6	35.0		37.0	32.2		32.6	33.0	
Former	48.3	41.8		51.1	42.8		46.0	52.2		48.3	50.6	
Current	23.0	16.4		15.4	22.2		17.0	15.6		19.1	16.4	
Alcohol past year (g/day)			0.20			0.86			0.15			< 0.001
No alcohol	25.5	25.8		18.3	17.9		20.0	16.7		21.8	17.3	
< 2.0	25.7	24.4		25.3	21.7		21.8	20.4		24.3	20.7	
2.0–4.99	29.1	19.7		25.6	28.3		28.2	27.5		27.9	27.2	
5.0–9.99	10.1	12.1		17.2	16.3		16.5	16.3		14.0	16.1	
≥ 10.0	9.6	18.2		13.6	15.8		13.5	19.2		11.9	18.7	
Diseases												
CVD	25.5	35.8	0.07	38.7	39.8	0.81	42.1	38.8	0.26	34.3	19.2	0.02
Diabetes	7.8	7.5	0.92	9.9	10.2	0.92	11.2	9.2	0.24	9.5	53.8	0.82
Chronic pain	34.8	26.9	0.20	38.7	37.6	0.82	43.5	36.4	0.02	38.6	27.0	0.19
BMI categories (kg/m^2^)			0.65			0.40			0.35			0.01
< 25	20.1	25.4		20.9	16.2		21.2	19.3		21.0	19.2	
25–29.9	45.3	40.3		48.9	49.7		50.7	55.1		48.0	53.8	
≥ 30	33.7	34.3		30.1	34.1		28.1	25.6		31.0	27.0	

*SD* standard deviation, *CVD* Cardiovascular disease (Myocardial infarction, angina pectoris, atrial fibrillation, heart (bypass) surgery, stent placement and/or use of antihypertensive drugs), *BMI* body mass index

^a^Subgroups may not total to this number due to missing values

^b^Municipalities included in winter, inland area: Kautokeino, Karasjok

^c^Municipalities included in winter, coast area: Tana and Nesseby

^d^Municipalities included in autumn/spring, coast area: Porsanger, Kåfjord, Storfjord, Lyngen, Skånland and Evenes

The distribution of PA levels among men and women overall showed that there was a higher proportion reporting low PA levels in the older age groups, independently of sex and ethnicity (Table 3). For Sami women overall, the mean PA level was lower compared to non-Sami women (5.2 versus 5.6), and this finding was more pronounced (4.9 versus 5.6) in the inland of Finnmark (Kautokeino/Karasjok) (Table 4). We found no statistically significant differences in mean self-reported PA in Sami and non-Sami men overall (Table 4). However, separated by geographical areas, Sami men situated at the Finnmark coastal areas (Tana/Nesseby) reported a statistically significantly higher PA compared to non-Sami men in the same area (5.3 versus 4.8).

**Table 3:** Physical activity level (%). The SAMINOR 2 Clinical Survey (2012–2014)

	Sami (*n* = 2307)						Non-Sami (*n* = 3321)						
	n	1–2	3–4	5–6	7–8	9–10	n	1–2	3–4	5–6	7–8	9–10	*P*-value
Women													
40–49 years	294	8.5	24.8	33.7	25.9	7.1	434	6.2	18.4	34.1	33.6	7.6	0.08
50–59 years	365	10.1	23.3	31.2	29.0	6.3	472	6.8	19.9	34.7	29.2	9.3	0.14
60–69 years	384	10.4	28.9	35.4	20.6	4.7	567	7.2	23.5	38.8	24.9	5.6	0.08
70–79 years	187	18.2	28.9	31.6	15.0	6.4	305	9.5	26.2	36.7	20.7	6.9	0.04
Total crude	1230	11.1	26.3	33.2	23.5	6.0	1778	7.3	21.8	36.2	27.4	7.3	< 0.001
Total age std.^a^	1230	11.1	26.3	33.2	23.4	6.1	1778	7.2	21.6	36.1	27.8	7.4	< 0.001
Men													
40–49 years	216	14.8	25.9	30.6	23.6	5.1	304	6.3	28.3	35.9	25.7	3.9	0.02
50–59 years	287	9.8	25.8	35.9	22.3	6.3	377	9.0	26.0	36.9	23.1	5.0	0.96
60–69 years	373	9.1	33.2	33.8	16.6	7.2	552	10.0	28.6	34.1	23.4	4.0	0.03
70–79 years	201	10.0	32.8	30.3	19.4	7.5	310	11.3	23.5	39.7	19.0	6.5	0.13
Total crude	1077	10.6	29.7	33.1	20.1	6.6	1543	9.3	26.9	36.2	22.9	5.5	0.03
Total age std.^a^	1077	11.0	29.1	32.9	20.5	6.4	1543	8.9	27.0	36.2	23.2	4.7	0.02

*std* standardised

^a^Direct standardisation to the age distribution (in 10-year age groups) of the invited sample. Ethnic differences are tested with the direct command tabi in Stata with the expected numbers in each cell as input, calculated from age-standardised proportions

**Table 4:** Mean physical activity level for Sami and non-Sami. The SAMINOR 2 Clinical Survey (2012–2014)

	Winter, inland^a^ (*n* = 1202)			Winter, coast^b^ (*n* = 964)			Autumn/spring, coast^c^ (*n* = 3462)			Total (*n* = 5628)		
	Sami	Non-Sami	*P*-value	Sami	Non-Sami	*P*-value	Sami	Non-Sami	*P*-value	Sami	Non-Sami	*P*-value
Women												
40–49 yr	5.4 (2.1)	5.8 (2.0)	0.47	5.5 (2.0)	5.8 (2.2)	0.43	5.5 (2.2)	5.9 (2.0)	0.08	5.4 (2.1)	5.9 (2.0)	0.01
50–59 yr	5.0 (2.2)	5.9 (2.1)	0.02	5.5 (2.3)	5.2 (2.2)	0.50	6.1 (1.9)	5.8 (2.1)	0.28	5.4 (2.2)	5.8 (2.1)	0.02
60–69 yr	4.7 (2.1)	5.3 (2.4)	0.16	5.4 (2.0)	5.0 (2.0)	0.26	5.5 (2.0)	5.5 (2.0)	1.0	5.1 (2.1)	5.4 (2.0)	0.01
70–79 yr	4.0 (1.9)	5.1 (2.4)	0.07	5.8 (2.4)	5.4 (2.2)	0.49	4.9 (2.5)	5.2 (2.1)	0.47	4.8 (2.3)	5.2 (2.1)	0.03
Total	4.9 (2.1)	5.6 (2.2)	0.002	5.5 (2.1)	5.3 (2.1)	0.41	5.6 (2.1)	5.6 (2.1)	0.79	5.2 (2.2)	5.6 (2.1)	< 0.001
Men												
40–49 yr	4.6 (2.3)	5.5 (1.6)	0.15	5.4 (1.7)	5.5 (1.7)	0.84	5.6 (2.3)	5.3 (1.9)	0.31	5.1 (2.2)	5.3 (1.9)	0.17
50–59 yr	5.0 (2.2)	4.2 (1.8)	0.12	5.5 (2.1)	4.6 (1.9)	0.02	5.6 (2.0)	5.5 (2.0)	0.64	5.3 (2.1)	5.3 (2.0)	0.93
60–69 yr	4.9 (2.2)	4.2 (1.8)	0.22	5.0 (2.0)	4.6 (1.8)	0.22	5.3 (2.0)	5.3 (2.0)	1.0	5.0 (2.1)	5.1 (2.0)	0.43
70–79 yr	5.0 (2.3)	5.8 (1.8)	0.21	5.5 (2.2)	4.8 (2.7)	0.26	4.7 (2.2)	5.2 (2.1)	0.12	5.1 (2.2)	5.2 (2.1)	0.63
Total	4.9 (2.2)	4.8 (1.9)	0.89	5.3 (2.0)	4.8 (2.0)	0.01	5.3 (2.1)	5.3 (2.0)	0.92	5.1 (2.2)	5.2 (2.0)	0.23

Note: Values are means (standard deviation)

*yr* years

^a^Municipalities included in winter, inland area: Kautokeino and Karasjok

^b^Municipalities included in winter, coast area: Tana and Nesseby

^c^Municipalities included in autumn/spring, coast area: Porsanger, Kåfjord, Storfjord, Lyngen, Skånland and Evenes

There were no differences in mean PA level between Sami and non-Sami men over different BMI categories (results not shown). For Sami women there was a lower PA level compared to non-Sami women within the normal weight and obese categories (results not shown). In total, the PA level decreased with higher BMI, independently of gender and ethnicity.

Results of the associations between ethnicity and PA level from adjusted linear regression models were consistent with the (unadjusted) results from two-sample t-tests of mean PA level, both for men and women (Table 5). However, in the fully adjusted analyses, the only statistically significant ethnic difference in PA level was observed for women when all regions were combined.

**Table 5:** Associations between Sami ethnicity and level of physical activity. The SAMINOR 2 Clinical Survey (2012–2014)

	Winter, inland^a^		Winter, coast^b^		Autumn/spring, coast^c^		Total	
	Model 1^d^	Model 2^e^	Model 1^d^	Model 2^e^	Model 1^d^	Model 2^e^	Model 1^d^	Model 2^e^
Women	*n* = 687	*n* = 585	*n* = 496	*n* = 456	*n* = 1825	*n* = 1722	*n* = 3008	*n* = 2763
Sami ethnicity	−0.74 (0.001)	−0.44 (0.08)	0.18 (0.35)	0.16 (0.39)	−0.05 (0.69)	−0.01 (0.92)	−0.37 (< 0.001)	−0.27 (0.001)
Men	*n* = 515	*n* = 473	*n* = 468	*n* = 435	*n* = 1637	*n* = 1553	*n* = 2620	*n* = 2461
Sami ethnicity	0.04 (0.89)	0.05 (0.86)	0.52 (0.006)	0.36 (0.06)	0.01 (0.95)	0.05 (0.71)	−0.1 (0.22)	−0.08 (0.32)

Note: Values are β coefficients from linear regression analyses with p-values in parentheses

^a^Municipalities included in winter, inland area: Kautokeino and Karasjok

^b^Municipalities included in winter, coast area: Tana and Nesseby

^c^Municipalities included in autumn/spring coast area: Porsanger, Kåfjord, Storfjord, Lyngen, Skånland and Evenes

^d^Model 1: adjusted for age

^e^Model 2: adjusted for age, education (< 13/≥13 years), smoking, alcohol intake (< 2, 2–4.99, 5–9.99, > 10 g/day), cardiovascular diseases (yes/no), diabetes (yes/no), chronic pain (yes/no) and body mass index (< 18.5, 18.5–24.9, 25–29.9, ≥30 kg/m^2^)

## Discussion

In this population of Sami and non-Sami adults in the rural northern part of Norway, the results showed that nearly 60% of the participants reported PA levels above five using a 10-point scale ranging from low to high PA. Overall, we found small differences in self-reported PA between the Sami and the non-Sami populations. Investigating the differences when stratifying by geographical areas, Sami men living in the Finnmark coast areas that were surveyed during winter (Tana and Nesseby) reported statistically significantly higher PA levels compared to non-Sami men in the same area. For Sami women, we observed lower PA levels overall compared to non-Sami women, and especially in women living in the inland of Finnmark (Kautokeino, Karasjok). However, these differences are small and in the adjusted models, a statistically significant ethnic difference was only observed when analysing all regions combined. Sami women with BMI in the normal or obesity range reported lower PA compared to non-Sami women within the same range.

The PA levels in the adult population in Norway from 2008/2009 showed that only 20% of the adult population reached the national recommendations of PA [7]. Furthermore, this did not differ between women and men, and the PA levels decreased after the age of 65 years [7]. In our study, we cannot differentiate whether the participants reached the national recommendations or not, as the scale does not allow for this interpretation. However, fewer participants reported PA levels in the upper levels of the scale, indicating that a low proportion of adults consider themselves as very physically active in total over leisure time, work and transportation. In addition, among the participants in our study, the PA levels decreased with increasing age in both women and men, independently of ethnicity.

In order to evaluate whether PA levels differ by ethnicity, there are few studies to compare with, as most studies have been conducted in the USA and ethnicity defined as “white” or “non-white, or broken down to “Hispanics”, “Asians”, “African Americans” [26]. The findings from most of these studies are weak or no association between PA levels and ethnicity, and some found inconsistent results [26]. Of the few studies describing PA levels in the Sami populations, the SAMINOR 1 Survey (2003–2004) found that Sami women had significantly lower levels of leisure-time PA than non-Sami women, as for men no ethnic differences were observed [16]. In the Finnmark County studies [21, 27, 28] conducted in the 1970’s and 1980’s, the participants reported work and leisure-time PA during the last year on a scale with four categories: sedentary, moderate, intermediate, and intensive [21]. Results showed that Sami women and men were more physically active at work compared to their non-Sami counterparts. In leisure-time, the Sami women were less active, while the Sami men had higher PA levels than their non-Sami counterparts did. However, combining work and leisure-time activity, a higher proportion of both Sami women and men were in the high physically active group, driven by work PA [21]. Indeed, census data from 1970 showed that the number working in or associated with primary industries – including Sami reindeer husbandry – was higher in Sami than in the non-Sami population. Whether this is the case today is, however, uncertain as updated data do not exits [29]. Nonetheless, results from qualitative interviews indicated that Sami reindeer herders and Sami and non-Sami farmers were active throughout the day and had no clear boundaries between work and leisure-time activity. Some Sami participants reported that fishing, hunting and berry-picking were perceived as something between work and leisure time activity [21]. This could influence the way especially Sami people interpret questions about physical activity. In these former studies, Sami ethnicity was defined differently than in our study, and none of them required both self-definition and a Sami language connection. The first study had a very wide definition of Sami ethnicity (ticking Sami in one of eleven questions regarding language, ethnic background and self-perceived ethnicity), while the other referred to having at least two grandparents of Sami origin [21, 27, 28]. In addition, the SAMINOR 1 Survey and the Finnmark County studies comprised participants in other municipalities and had other measures of physical activity than the SAMINOR 2 did. For these reasons, it is challenging to compare the PA levels between the different studies. To our knowledge, there are no other published studies on PA levels conducted in rural areas inhabited with Sami population.

This study had both strengths and limitations. The response rate in SAMINOR 2 was modest (48%) and varied between municipalities, from 41% in Evenes to 56% in Kautokeino [22]. Women participated to a stronger degree than men and participation rates increased with increasing age [22]. We have no information about ethnic affiliation in national registries due to legislation. However, the 11 different questions about ethnic affiliation includes both objective and subjective criteria and ensure the best way to categorize ethnicity. No ethnic differences were found in non-response in a selected sample of the SAMINOR 2 Questionnaire Survey, when using ethnicity information collected in the SAMINOR 1 Survey [30]. Underreporting of Sami ethnicity is likely. The historical forced assimilation policy and experiences of stigmatisation and discrimination may have made some Sami individuals reluctant to reveal their Sami background. We assume that this misclassification of ethnic background is non-differential, and that the differences in physical activity between Sami and non-Sami may be underestimated.

PA level was assessed by self-report on an ordinal scale of 1 to 10 and refers to the total amount of PA across different domains, including recreation, occupation, transportation, and household in one global score. This scale cannot differentiate between intensity, duration, and frequency of PA, nor the type of PA and the differences in the perception of the scale. Therefore, the use of a global scale on PA level does not allow for direct comparison with other measurements of PA, hence using self-report of PA, measurements errors cannot be ruled out. However, the assessment of PA levels among adult Norwegian women in the Norwegian Women and Cancer study, have shown this scale is able to range the PA levels as exposure investigating premature mortality, cardiovascular deaths and cancer deaths, and risk of several cancers, i.e. breast, colorectal, endometrial and lung cancer [24, 31–37]. A limitation of this study is that the scale is not validated among women outside this age span, among men, people of Sami ethnicity, or people living in rural areas of Northern Norway. The PA scale has no pre-defined reference for each value of the numbers from 1 to 10, making it difficult to describe the true level of PA.

In a cross-sectional design, we were not able to observe the change of a modifiable behaviour as PA level and we cannot describe any trends. In the northern parts of Norway, there are also considerable seasonal variations, which may have resulted in biased estimates of the PA levels across the different geographical areas. Therefore, we stratified our analysis to the different geographical areas as the data collection took place at different seasonal periods for each municipality. In addition, the areas differ considerably by ethnicity. Sami and non-Sami populations within the same area and time of the year for data collection were compared, which ensured that the ethnic comparisons were not affected by differences in weather conditions, access to sports facilities etc. However, this resulted in a low sample size in each region. Furthermore, we could not measure the impact of any seasonal variations in this cross-sectional design, which makes it impossible to conclude whether the seasons have had an impact on the PA levels in our study. However, the general impact of seasonal changes on PA levels are inconsistent and the results are conflicting in the magnitude of the variation on PA levels [38–40]. Studies have reported that other predictors of PA are important in influencing the PA levels among adults, like sociodemographic characteristics, the perception of barriers that hinder PA (i.e. motivation, lack of time, social support, resources) and health behaviours (i.e. smoking, anthropometry and diet) [26]. Furthermore, a stable pattern of daily life behaviours is perhaps more important influencing PA levels as daily activities competes in time spent within a day (e.g. sleep, leisure, occupation, transportation and home) [41].

## Conclusion

This study showed small differences in PA levels between Sami and non-Sami men, although in Tana and Nesseby, PA levels were slightly higher in Sami men compared to non-Sami men. The Sami women reported lower PA levels compared to their non-Sami counterparts, driven by the results in the Sami dominant municipalities of Karasjok and Kautokeino. This study indicates that it is important to identify whether there are differences in PA levels in various ethnic populations, together with other predictors for PA in future planning of public health interventions.

## Data Availability

The data that support the findings of this study are available from the SAMINOR Study (www.saminor.no). Restrictions apply to the availability of these data, which were used under license for this study. Data are available upon reasonable request to the SAMINOR Project Board and with permission of the Regional Committee for Medical and Health Research Ethics.
